# Extracellular Vesicle-Mediated Delivery of Antioxidant Enzymes: Emerging Insights and Translational Opportunities

**DOI:** 10.3390/antiox14121504

**Published:** 2025-12-14

**Authors:** Junyu Wang, Yakun Li, Robin P. F. Dullaart, Peter Olinga, Han Moshage

**Affiliations:** 1 Department of Gastroenterology and Hepatology, University Medical Center Groningen, University of Groningen, 9700 RB Groningen, The Netherlands; j.wang03@umcg.nl (J.W.); y.li01@umcg.nl (Y.L.); 2 Department of Pharmaceutical Technology and Biopharmacy, Groningen Research Institute of Pharmacy, University of Groningen, 9700 RB Groningen, The Netherlands; p.olinga@rug.nl; 3 Department of Internal Medicine, University Medical Center Groningen, University of Groningen, 9700 RB Groningen, The Netherlands; dull.fam@12move.nl

**Keywords:** extracellular vesicles (EVs), antioxidant enzymes (AOEs), oxidative stress, redox signaling, intercellular communication, therapeutic delivery

## Abstract

Oxidative stress is a key contributor to the onset and progression of diverse pathological conditions, including metabolic dysfunction-associated steatotic liver disease (MASLD), neurodegeneration, cardiovascular disorders, and cancer. Conventional antioxidant therapies, such as small-molecule scavengers or systemic enzyme administration, are limited by poor stability, inefficient delivery, and off-target effects. Extracellular vesicles (EVs), particularly exosomes, are increasingly recognized as natural carriers of antioxidant enzymes (AOEs), including catalase, superoxide dismutases, glutathione peroxidases, peroxiredoxins, and thioredoxin. These vesicles not only protect enzymes from degradation but also enable targeted delivery to recipient cells, where they can actively modulate redox homeostasis. In this review, we summarize current evidence for AOEs as bona fide EV cargo, outline mechanisms that govern their selective packaging and transfer, and highlight their roles in intercellular communication under physiological and pathological conditions. We also discuss emerging therapeutic applications of both natural and engineered EVs for redox modulation, along with the challenges of quantifying enzymatic activity, ensuring reproducibility, and scaling clinical translation. By integrating insights from cell biology, redox signaling, and translational research, we propose that EV-mediated AOE delivery represents a promising next-generation strategy for combating oxidative stress-related diseases.

## 1. Introduction

Reactive oxygen species (ROS) are continuously generated as byproducts of normal cellular metabolism and signaling [1]. Under physiological conditions, redox homeostasis is tightly regulated by antioxidant defense systems composed of enzymatic and non-enzymatic components [1,2]. However, excessive ROS production or impaired antioxidant defense capacity leads to oxidative stress, a critical factor implicated in the onset and progression of various diseases, including metabolic dysfunction-associated steatotic liver disease (MASLD), cardiovascular disorders, neurodegenerative diseases, and cancer [3,4,5,6]. In these contexts, oxidative stress not only induces direct macromolecular damage but also acts as a signaling hub that exacerbates inflammation, apoptosis, and cellular senescence, thereby perpetuating tissue dysfunction [3,7,8].

Over the past decades, antioxidant therapies have been extensively explored to mitigate oxidative damage [4,9,10]. Classical strategies, such as small-molecule scavengers (e.g., N-acetylcysteine, vitamin E, vitamin A) or systemic administration of antioxidant enzymes (AOEs) like catalase and superoxide dismutase (SOD), have demonstrated limited clinical efficacy due to poor bioavailability, short half-life, and inadequate tissue targeting [11,12]. Additionally, nonspecific systemic delivery may disrupt physiological redox signaling, highlighting the need for more selective and efficient approaches to restore redox balance in diseased tissues [10,12].

Extracellular vesicles (EVs) are lipid bilayer-enclosed nanoparticles secreted by virtually all cell types and have emerged as promising natural carriers for therapeutic cargo [13,14,15]. Their intrinsic stability, low immunogenicity, and cell type-specific tropism give EVs clear advantages over synthetic nanocarriers for delivering bioactive molecules [15]. Recent studies have revealed that EVs can encapsulate a range of AOEs, including catalase, SODs, glutathione peroxidases (GPXs), peroxiredoxins (PRDXs), and thioredoxin (TXN), either passively reflecting cellular content or through selective sorting mechanisms. Once delivered to recipient cells, these enzymes can modulate local redox homeostasis, influence signaling cascades, and even alter disease trajectories [1,10,16,17,18,19].

This review aims to provide a comprehensive overview of current evidence for AOEs as bona fide EV cargo, emphasizing the mechanisms underlying their selective packaging, secretion, and functional transfer. We further discuss how EV-associated AOEs contribute to intercellular redox communication under physiological and pathological conditions. Finally, we explore translational strategies using natural and engineered EVs for therapeutic redox modulation, while addressing challenges in enzyme quantification, reproducibility, and clinical scalability. Through this integrative perspective, we aim to elucidate how EV-mediated AOEs delivery may offer a next-generation paradigm for combating oxidative stress-related diseases.

## 2. Antioxidant Enzymes (AOEs) in EVs

**Figure 1:**
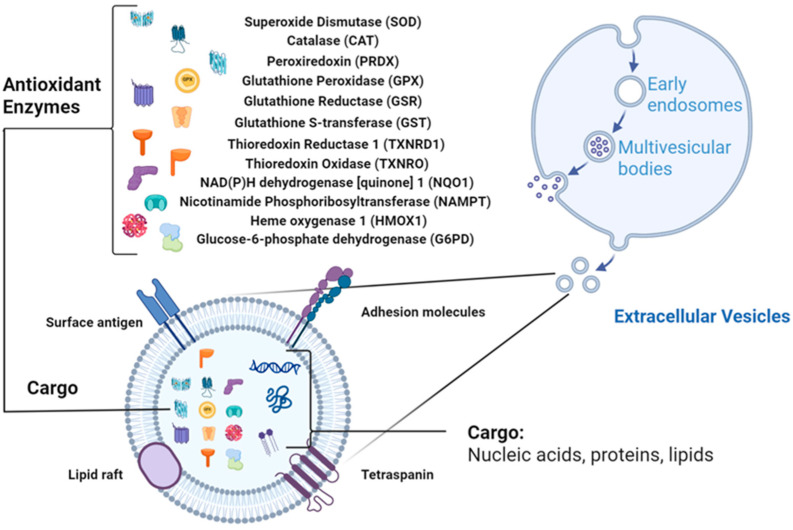
EV structure with antioxidant enzymes (AOEs) as cargo (Created in BioRender. Wang, J. (2026) https://BioRender.com/t0f0rdi, accessed on 10 October 2025).

EVs are increasingly recognized as carriers of redox-regulatory enzymes that actively shape intercellular oxidative balance. Proteomic and biochemical evidence across multiple species show that AOEs are present in EVs and retain catalytic activity upon transfer to recipient cells (Figure 1). The following sections summarize major antioxidant enzyme families identified in EVs, drawing upon curated data from PubMed, ProteomeXchange, Vesiclepedia, and ExoCarta, and highlight representative findings summarized in Table 1.

### 2.1. Superoxide Dismutases (SOD1/2/3)

Superoxide dismutases (SODs) catalyze the conversion of superoxide (O_2_^−^•) into hydrogen peroxide (H_2_O_2_) and oxygen, forming the first enzymatic defense line against ROS. All three major isoforms, cytosolic SOD1, mitochondrial SOD2, and extracellular SOD3, have been consistently detected in EVs from stem cells, adipose tissue, skeletal muscle, macrophages, and plasma [17,19,20,21,22,23,24,25,27,28,29,30,33,34,35,36]. Multiple studies confirm that EV-associated SODs retain catalytic activity, enabling functional delivery to recipient cells [16,17,25,27,28,30,33,35,36]. Exercise markedly increases SOD enrichment in circulating EVs, particularly those originating from contracting skeletal muscle, thereby enhancing systemic oxidative stress resistance and contributing to cardioprotection and neuroprotection [24,35]. Genetic overexpression of SOD3 in MSCs further augments the antioxidative and immunomodulatory effects of their secreted EVs, demonstrating that vesicular SOD3 can be functionally potentiated for therapeutic use [30]. EVs from several plant sources also contain active SODs, expanding the diversity of biological systems in which SOD loading occurs [26,37,38,39,40].

### 2.2. Catalase

Catalase decomposes H_2_O_2_ into water and molecular oxygen, preventing toxic H_2_O_2_ accumulation [51]. It is a frequent and functionally validated component of EVs from plasma, cardiac tissue, adipose-derived vesicles, and macrophages [17,20,22,23,24,27,29,34,36,41,52]. Catalase-loaded EVs protect hepatocytes and cardiomyocytes from oxidative injury and appear dynamically regulated by physiological states, for example, exercise increases both catalase abundance and activity in circulating EVs [41]. Catalase is also present in bacterial and plant-derived EVs, where it can modulate host or environmental redox states, for instance, KatB-containing bacterial EVs suppress ROS in plant tissues to promote pathogen survival [26,38,39,42].

### 2.3. Peroxiredoxins (PRDXs)

PRDXs (1–6) reduce H_2_O_2_ and organic hydroperoxides via the thioredoxin system. Multiple PRDX isoforms have been identified in plasma- and stem cell-derived EVs, and mitochondrial-derived vesicles [17,19,23,24,25,34,43,44,45,46]. PRDX abundance within EVs varies with external stimuli, indicating redox-responsive packaging, although the enzymatic activity of EV-associated PRDXs is less frequently verified experimentally [19,45].

### 2.4. Glutathione Peroxidases (GPXs)

GPXs 1/3/4 reduce H_2_O_2_ and lipid hydroperoxides using glutathione (GSH) as a cofactor. GPX proteins and activity have been detected in EVs from stem cells, cardiac tissue, adipose tissue, and plasma [20,22,24,36,47,48]. GPX-positive EVs protect recipient cells against oxidative and lipid peroxidation-associated damage [36]. Interestingly, GPX loading into adipose-derived EVs appears selectively regulated, as high-fat diet increased catalase but not GPX activity [36].

### 2.5. Glutathione System Enzymes and Glutathione S-Transferase (GST)

Key glutathione cycle enzymes, including glutathione reductase (GSR) and glutathione S-transferase (GST), are present in plasma- and cardiac-derived EVs [21,22,24,41]. The co-presence of these enzymes suggests that EVs may operate as self-contained glutathione-regenerating units, sustaining redox cycling and enhancing antioxidant capacity in recipient cells.

### 2.6. Thioredoxin (TXN) and Related Enzymes

Thioredoxin (TXN) and thioredoxin reductase (TXNRD1), central mediators of thiol redox balance, have been consistently identified in plasma- and mitochondrial-derived EVs [16,17,24,25,27,41]. Exercise enhances TXN abundance in circulating EVs, indicating that vesicular thiol-reducing capacity contributes to systemic adaptation to oxidative challenge [16,24,27,41]. These findings support a functional role for EV-associated TXN in intercellular redox regulation under both homeostatic and stress conditions.

### 2.7. Additional Redox-Active Enzymes

Several other redox-modulating enzymes have been identified in EVs, including NAD(P)H dehydrogenase [quinone] 1 (NQO1), nicotinamide phosphoribosyltransferase (NAMPT), heme oxygenase 1 (HMOX1), and glucose-6-phosphate dehydrogenase (G6PD) [17,21,24,35,41,49,50]. These enzymes are functionally linked to NAD(P)H regeneration, quinone detoxification, and heme catabolism, thereby connecting vesicular redox buffering to broader metabolic and cytoprotective pathways.

### 2.8. Functional Evidence of Enzymatic Activity

Beyond proteomic and Western blotting identification, several studies have provided functional evidence that EV-associated AOEs retain catalytic activity after secretion. Catalase- and GPX-containing EVs from adipose tissue, as well as SOD-enriched vesicles released from human neutrophils, exhibit measurable enzymatic activity in standard redox assays [33,36]. SOD-loaded EVs from human umbilical cord MSCs (hUC-MSCs) effectively alleviate hepatic ischemia–reperfusion injury in rats by reducing oxidative stress and attenuating neutrophil-mediated inflammation [28]. Plasma-derived EVs released during exercise or inflammatory stimulation show sustained GSR, catalase, and NAMPT activity, contributing to tissue protection and systemic redox buffering [41,49]. Comparable catalytic activities have also been documented in plant-derived EVs, particularly for SOD and catalase [37,38,39,40]. Collectively, these findings confirm that EVs function as active vectors of antioxidant enzymatic activity, shaping redox signaling and enhancing cytoprotection in recipient cells.

## 3. Mechanisms of Antioxidant Enzymes (AOEs) Packaging and Transfer

**Figure 2:**
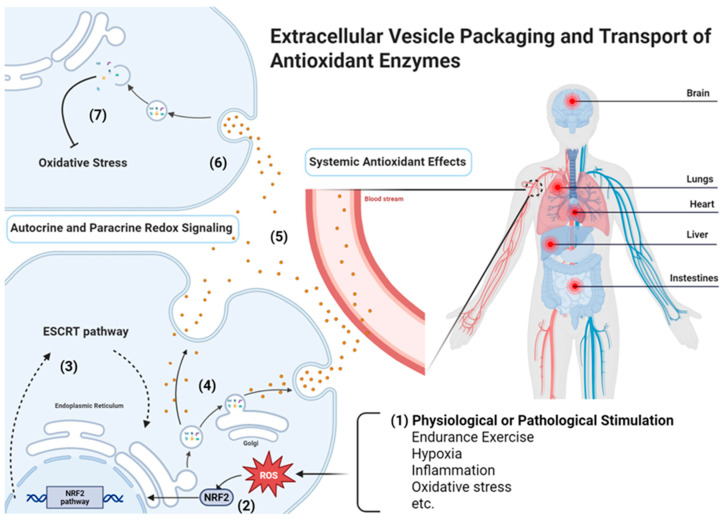
Mechanisms of antioxidant enzymes (AOEs)-loading EV packaging and delivery (Created in BioRender. Wang, J. (2026) https://BioRender.com/t0f0rdi, accessed on 10 October 2025). EV-mediated antioxidant defense involves several coordinated steps: (1) Cargo incorporation occurs either passively under basal conditions or is enhanced by stress-induced enrichment; (2) Redox-related signaling pathways, such as the Nrf2 pathway, regulate the expression and sorting of AOEs; (3) Endosomal sorting can proceed through the endosomal sorting complex required for transport (ESCRT)-dependent or ESCRT-independent mechanisms, involving tetraspanins and ceramide-mediated pathways; (4) AOEs (e.g., catalase, SOD, GPX) are selectively packaged into EVs; (5) EVs are released and transported locally via autocrine and paracrine redox signaling or systemically through circulating EVs that mediate organism-wide redox adaptation; (6) Recipient cells internalize EVs through receptor-mediated uptake, endocytosis, or membrane fusion; (7) The delivered AOEs restore redox homeostasis and exert protective effects within recipient cells.

The selective incorporation and intercellular transfer of AOEs via EVs are key determinants of their functional impact. Although the underlying mechanisms are not fully understood, evidence suggests that both canonical vesicle biogenesis pathways and redox-dependent regulatory processes influence the loading, release, and uptake of enzymatically active antioxidant cargo (Figure 2). While AOE-containing EVs have also been reported in plants and bacteria, this review focuses on the mechanisms of AOE packaging and transfer in human and animal physiological and pathological contexts.

### 3.1. Cellular Origins of Antioxidant Enzymes (AOEs) in EVs

The origin of AOE-containing EVs varies depending on the physiological context and the cell types involved. Diverse AOEs have been identified in EVs secreted by multiple cell types, including stem cells, immune cells, and cardiomyocytes (Table 1), supporting the notion that EV-mediated redox modulation is a conserved intercellular mechanism. To date, however, no study has conclusively demonstrated that specific AOEs are selectively packaged into EVs by defined cell types or under strictly controlled conditions. Instead, most available evidence highlights dynamic changes in AOE abundance within EVs in response to physiological or pathological stimuli (Table 1). Under homeostatic conditions, metabolically active tissues such as skeletal muscle and heart continuously release low levels of AOE-loaded EVs [22,35], and EV-derived AOEs further increase following exercise training [41]. In contrast, during oxidative or inflammatory stress, immune cells, cardiomyocytes, and adipocytes become major contributors to the circulating EV pool [9,19,25,41,49]. These stress-responsive cells upregulate vesicle biogenesis and selectively enrich antioxidant cargo, thereby coupling intracellular redox dynamics to systemic oxidative balance.

### 3.2. Passive Incorporation Versus Stress-Induced Enrichment

AOEs loading into EVs can occur passively, reflecting the intracellular redox state, or actively in response to physiological stress. Exercise, hypoxia, and inflammation cues commonly increase EV secretion and modify their redox cargo. Conversely, severe oxidative or metabolic stress may reduce AOE content per vesicle, suggesting altered loading efficiency. Notably, EV-associated AOEs can increase following exercise training. Sagini et al. reported that elevated TXN in plasma EVs from endurance-trained females following acute exercise [16]. Exercise also enhances NAMPT release in circulating EVs, influencing NAD^+^ metabolism in recipient cells [49], and stimulates Nrf2-dependent antioxidant gene expression that becomes enriched in circulating EVs [35]. Although multiple studies have documented stress-related changes in AOE content in EVs from skeletal muscle, adipose tissue, and heart, the precise cellular origins of these EVs remain incompletely defined. Immune cells also actively secrete AOE-rich EVs. Iversen et al. showed that SOD3 is stored in secretory vesicles of neutrophils and released upon stimulation [33]. Likewise, macrophage-derived EVs deliver PRDX6 to tumor cells, promoting ferroptosis resistance [46]. Stem cell-derived EVs contain multiple AOEs even without external stimulation [19,20,28,30,44], suggesting a constant baseline source of circulating antioxidant activity. Collectively, current evidence supports that stress-induced AOE enrichment in EVs represents an adaptive mechanism to enhance antioxidant defense during periods of elevated oxidative load.

### 3.3. Sorting Pathways Influencing Antioxidant Enzymes (AOEs) Loading

Immune and stem cells can markedly increase AOE loading or the number of AOE-rich EVs under oxidative stress, reflecting both passive and active sorting mechanisms. AOE incorporation into EVs can be passive or stress-driven. Oxidative stress-responsive pathways, particularly Nrf2, influence EV cargo composition. Gao et al. proposed that Nrf2 activation upregulates cytoprotective proteins, including AOEs, which are subsequently released via EVs and transferred to distant tissues [35]. Although direct mechanistic studies remain limited, other stress-related pathways such as HIF-1, MAPK, and NF-κB may also contribute [2,35]. Mechanisms governing selective AOE packaging and the fate of EV-delivered AOEs remain unclear and represent a major knowledge gap. EV biogenesis involves multiple routes. The ESCRT-dependent pathway recruits specific cytosolic proteins, including potentially redox-active enzymes, into intraluminal vesicles via components such as TSG101, ALIX, and VPS4 [53,54,55,56]. ESCRT-independent mechanisms, including ceramide-driven budding and tetraspanin-enriched microdomains (CD9, CD63, CD81), may also support AOE incorporation [57,58]. The cellular redox state may further modify sorting efficiency, as oxidative stress can alter membrane lipid composition and induce post-translational modifications (e.g., S-glutathionylation, nitrosylation) that influence AOE localization and vesicular packaging [59,60].

### 3.4. Uptake Mechanisms in Recipient Cells

Upon release, EVs interact with recipient cells through multiple entry routes, including clathrin- or caveolin-mediated endocytosis, macropinocytosis, phagocytosis, receptor-mediated internalization, or direct membrane fusion [61,62]. Despite significant progress in elucidating the molecular mechanisms underlying EV uptake, the question of how specificity in EV–cell interactions are achieved remains largely unresolved. The route of uptake is influenced by factors such as vesicle size, lipid composition, and the presence of specific surface ligands [61,62,63]. For instance, tetraspanins (e.g., CD9, CD63, CD81) and integrins on EV membranes can mediate docking and internalization by hepatocytes, cardiomyocytes, or endothelial cells [15,61,63]. In particular, no studies to date have clearly identified the specific target cell types for AOE-enriched EVs or elucidated the precise uptake mechanisms by which these vesicles are internalized by recipient cells.

### 3.5. Functional Evidence of Enzyme Transfer

Growing functional evidence shows that EV-mediated transfer of AOEs can directly modulate redox status in recipient cells. Catalase-rich EVs from adipose tissue reduce intracellular ROS levels in hepatocytes and improve cell survival during oxidative stress, demonstrating that vesicle-delivered catalase remains enzymatically active after uptake [36]. Similarly, AOE-enriched EVs reduced oxidative damage in human iPSC-derived cardiomyocytes at baseline and after H_2_O_2_ exposure, producing a pronounced cardioprotective effect [41]. SOD-loaded EVs from stem cells exert pronounced antioxidant and anti-inflammatory effects by catalyzing superoxide dismutation and limiting oxidative tissue injury [30]. EV-associated TXN further protects cardiomyocytes from H_2_O_2_-induced oxidative stress and helps preserve thiol–redox balance during exercise- or inflammation-associated conditions [16]. These findings collectively indicate that EVs act as functional vectors of antioxidant capacity, enabling intercellular redox coordination and metabolic resilience.

### 3.6. Outstanding Questions and Knowledge Gaps

Despite these advances, several unresolved questions limit mechanistic insight. It remains unclear whether AOEs are selectively packaged into EVs via defined sorting signals or are incorporated passively based on cellular abundance. The contribution of post-translational modifications, protein–lipid interactions, or redox-sensitive motifs to sorting specificity also requires clarification. In recipient cells, the stability, trafficking routes, and catalytic lifespan of EV-derived enzymes, particularly within endosomal or lysosomal compartments, are still poorly characterized. Addressing these gaps will be essential to harness EV-mediated antioxidant delivery for therapeutic applications, enabling controlled modulation of redox signaling and improved translational reproducibility.

## 4. Functional Roles of EV-Derived Antioxidant Enzymes (AOEs)

EVs carrying AOEs exert multifaceted effects that extend beyond intracellular redox regulation, influencing both local microenvironments and systemic physiological processes. Through the transfer of catalytically active enzymes, EVs can buffer oxidative stress, modulate cell signaling, and shape disease outcomes across various biological contexts (Figure 2).

### 4.1. Autocrine and Paracrine Redox Signaling

EV-derived AOEs modulate redox homeostasis in neighboring or originating cells by regulating extracellular and intracellular ROS levels. Stem cell- and tumor-derived EVs can reduce oxidative burden in target cells [21,44,48]. Liu et al. showed that stem cell-derived EVs alleviate senescence by reducing oxidative stress, restoring mitochondrial function, and enhancing proliferation [44]. Similarly, hypoxic glioblastoma cells release GPx1-enriched EVs that buffer H_2_O_2_ in surrounding tumor cells, illustrating EV-mediated autocrine and paracrine protection [48]. In the cardiac microenvironment, EVs from cardiac stromal cells or MSCs deliver catalase and GPX to protect cardiomyocytes from oxidative injury [28,64]. Neutrophil-derived EVs containing SOD modulate cytokine responses and help limit inflammation [31,33]. In the central nervous system, glia-derived EVs reduce neuronal ROS and protect against oxidative and mitochondrial dysfunction [65,66]. In the liver, EV-mediated redox signaling may influence hepatocyte stress responses, hepatic stellate cell (HSC) activation, fibrosis progression, and MASLD pathogenesis [13,67,68,69,70,71,72,73]. AOE-enriched EVs can protect hepatocytes from H_2_O_2_- or lipotoxicity-induced apoptosis [36], and reduce ROS accumulation in liver endothelial cells [31]. Given that oxidative stress drives both steatosis and fibrogenesis, EV-based AOE transfer may function as an intrinsic mechanism limiting HSC activation and fibrotic remodeling. Clarifying these EV-mediated redox interactions may reveal new therapeutic opportunities for restoring redox balance in chronic liver disease.

### 4.2. Systemic Antioxidant Effects

Circulating EVs also serve as vehicles for systemic redox adaptation. Exercise-induced EVs enriched in SOD, catalase, NAMPT, and TXN contribute to whole-body oxidative stress resistance by transferring antioxidant capacity to distant tissues such as the myocardium and central nervous system [16,35,41]. Abdelsaid et al. demonstrated that exercise improves the angiogenic function of circulating exosomes in type 2 diabetes by enriching them with extracellular SOD3; these SOD3-loaded exosomes attenuate oxidative stress, restore endothelial nitric oxide signaling, and promote vascular repair [32]. Bao et al. similarly reported that neutrophils alleviate sepsis-associated coagulopathy by releasing EVs enriched with mitochondrial SOD2, which reduce oxidative stress and endothelial injury, thereby preserving vascular integrity in a murine model of lipopolysaccharide (LPS)-induced sepsis [31]. Other circulating EV populations carrying AOEs have likewise been shown to confer antioxidant protection in recipient cells, contributing to systemic defense against oxidative stress [16,35,45,49]. Stem cell-derived EVs containing multiple AOEs effectively reduce ROS accumulation and improve metabolic recovery in models of ischemia, inflammation, and aging [28,30,44]. These findings highlight that EV-mediated enzyme transfer constitutes a physiological mechanism for inter-organ redox communication and systemic cytoprotection.

## 5. Therapeutic Applications

### 5.1. Natural EVs

#### 5.1.1. Stem Cell-Derived EVs

Stem cell-derived EVs have shown remarkable potential in protecting cells from oxidative stress, primarily due to the presence of AOEs. For instance, EVs from hUC-MSCs alleviated hepatic ischemia-reperfusion injury by reducing oxidative stress and inflammatory responses, with key AOEs like SOD playing a vital role [28]. Similarly, stem cell-derived EVs have been shown to improve aging cellular phenotypes, largely due to the abundance of AOEs they carry [44]. These findings highlight the therapeutic potential of stem cell-derived EVs, with AOEs being a critical component in mitigating oxidative stress.

In addition to their inherent antioxidant properties, the production of AOEs within stem cell-derived EVs can be further enhanced through various methods. For example, MSCs preconditioned with LPS, which increase the release of small EVs that effectively treat periodontitis through ROS-mediated antioxidant effects [21]. Additionally, overexpressing AOEs in MSCs can enhance the immunomodulatory abilities of their derived EVs, improving their therapeutic efficacy in models of inflammation and oxidative stress [30]. Moreover, hypoxic preconditioning of hUC-MSCs increases the expression of exosomal TXN-1, which has been shown to inhibit ferroptosis in doxorubicin-induced cardiotoxicity through mTORC1 signaling [74]. Quercetin, a natural antioxidant, enhances the antioxidant capacity of stem cells and their EVs by upregulating SOD1, thereby boosting their anti-inflammatory and antioxidant properties [19]. Developing strategies to enhance the antioxidant capacity of stem cell-derived EVs will improve their effectiveness in treating oxidative stress-related diseases.

#### 5.1.2. Immune Cell-Derived EVs

In addition to stem cells, immune cells represent another natural source of EVs rich in AOEs. Proteomic analyses have identified TXN II within dendritic cell-derived exosomes, suggesting its functional contribution to oxidative balance [75]. Extracellular SOD is stored in secretory vesicles of neutrophils and released into the extracellular space upon cellular activation [33]. In a mouse model of sepsis, circulating neutrophils secrete mitochondria-containing EVs highly enriched with SOD2 [31]. These vesicles effectively reduce endothelial ROS accumulation and alleviate disseminated intravascular coagulation [31]. Although direct evidence is limited, macrophage-derived exosomes are often engineered to enhance antioxidant properties, highlighting their potential as ROS-scavenging carriers for inflammatory disease treatment, tissue repair, and oxidative stress mitigation. Overall, the abundance of AOEs in immune cell-derived EVs likely reflects the activation and oxidative state of parent cells, offering insights for disease monitoring.

#### 5.1.3. Plant-Derived EVs

In recent years, plant-derived EVs have attracted growing attention due to their excellent biocompatibility and distinctive bioactivity, particularly in antioxidant and anti-inflammatory applications [76]. Numerous studies have identified high levels of key antioxidant enzyme families in plant-derived EVs isolated from sources including *Perilla frutescens*, various vegetables and fruits, *Aloe vera* peels, and microalgae [26,37,38,39,40,76,77]. These findings strongly support that AOEs are integral to the protein cargo of plant-derived EVs, providing a molecular foundation for their antioxidant functionality. These EVs are also widely used as delivery systems due to their low cost and abundant availability [78]. Nevertheless, evidence regarding the effective delivery of these enzymes to recipient cells remains limited, and their specific contribution to the overall antioxidant capacity of plant-derived EVs is still unclear.

#### 5.1.4. Milk-Derived EVs

Milk is a complex biological fluid containing various endogenous antioxidant enzymes, such as SOD, catalase, and GPX [79]. These enzymes are essential for neutralizing free radicals, protecting milk lipids from oxidative damage, and maintaining the nutritional and sensory quality of dairy products. Recent studies indicate that milk-derived EVs (MEVs) remain structurally stable under simulated gastrointestinal conditions [80,81] and exhibit antioxidant properties, including the ability to neutralize ROS and reduce oxidative stress [82,83]. However, research on the antioxidant function of MEVs is still limited. There is a lack of proteomic validation of the vesicle cargo and uncertainty about the localization of antioxidant enzymes, whether inside the vesicles or on their surface [84]. Future research should focus on providing direct and quantitative data to confirm the role of MEVs as natural carriers of antioxidant enzymes.

### 5.2. Engineered EVs

The use of engineered EVs containing AOEs has emerged as a promising strategy in modern therapeutics [85]. These natural EVs can be modified and enriched with AOEs using multiple engineering strategies. Common post-isolation loading techniques include sonication, electroporation, and mechanical extrusion. In parallel, genetic modification of donor cells represents an equally important strategy: by overexpressing specific AOEs in the parental cells, the intracellular abundance of these enzymes is increased, resulting in their efficient and physiologically compatible incorporation into secreted EVs [30]. This overexpression-based method avoids harsh physical manipulation and enables more controlled enrichment of therapeutic enzymes within EVs. Engineered EVs exhibit potent antioxidant effects, reducing oxidative stress, lipid peroxidation, and neuroinflammation, while also restoring mitochondrial function. They have demonstrated therapeutic potential in various disease models, especially for cardiovascular disease, cancer, and neurodegenerative disorders [86,87,88]. The key benefit of engineering EVs lies in their ability to enhance the natural properties of EVs, such as improved targeting capabilities and biocompatibility, and the ability to carry a variety of therapeutic payloads while minimizing potential off-target effects, which is a significant advantage over traditional drug delivery systems [89]. Table 2 summarizes the representative engineered AOE-loading EVs, including those generated by AOE overexpression in donor cells and those produced through exogenous enzyme encapsulation strategies. Despite their promise, important questions remain regarding the long-term safety, biodistribution, and potential immunogenicity of engineered EVs. As research advances, engineered EVs containing AOEs show great promise as a next-generation therapeutic platform.

### 5.3. Nanozymes

Unlike natural AOEs encapsulated in EVs, nanozymes are inorganic or nanostructured catalysts that functionally imitate AOE activity but are not biological proteins. As shown previously, natural AOEs rely on defined amino-acid sequences and metal/cofactor-based active centers, and their activity is highly specific, regulated by cellular context, and subject to denaturation or proteolytic degradation. In contrast, nanozymes generate enzyme-like effects through surface redox reactions, catalytic metal centers, or defect-rich lattices. Their activity is often broader and less substrate-selective, but it is more resistant to harsh chemical or thermal conditions [95,96]. These nanozymes offer promising alternatives to traditional enzyme therapies, as summarized in several reviews [97,98,99,100]. The most common antioxidant nanozymes are metal oxide nanoparticles (CeO_2_, MnO_2_, Fe_3_O_4_), which can mimic SOD, catalase, and even PRDX activities [101]. Single-atom catalysts, through precise design of their metal centers, can mimic several natural AOEs simultaneously, making them a current research hotspot [102]. Carbon-based nanozymes and metal–organic framework (MOF)-based nanozymes, with adjustable surface functional groups and pore structures, can perform multiple antioxidant functions and are applicable in biosensing, drug delivery, and disease treatment [103,104,105]. Overall, nanozymes complement natural AOEs by providing high stability, scalable synthesis, and tunable catalytic performance, while EV-delivered natural enzymes offer higher biological specificity and compatibility. Combining these two strategies may therefore yield synergistic antioxidant therapies.

### 5.4. Diagnostic Value of Antioxidant Enzymes (AOEs) in EVs

EVs are increasingly recognized as promising diagnostic tools because they function as a “liquid biopsy” that reflects physiological and pathological status. The content of EVs, including AOEs, can provide valuable insights about disease mechanisms. In amyotrophic lateral sclerosis (ALS), for example, SOD1 found in circulating EVs has been proposed as a potential biomarker, offering diagnostic and prognostic value for ALS progression [106]. Similarly, in echinococcosis, a parasitic infection caused by *Echinococcus* species, high levels of TXN in EVs serve as an early diagnostic indicator, detectable as early as 10 days post-infection [107]. Moreover, mitochondria-derived EVs also carry enzyme cargo that may be implicated in diseases associated with mitochondrial dysfunction [25]. These EVs, particularly under oxidative stress, contain key AOEs and redox-active proteins, which could offer diagnostic value for early detection and monitoring of disease progression in mitochondrial disorders or diseases related to oxidative stress, like cardiovascular diseases or neurodegenerative diseases. Together, these findings highlight the potential of EV cargo profiling as a non-invasive approach for early detection and disease monitoring, although further validation is still required.

## 6. Challenges and Limitations

EV-based delivery of AOEs holds significant promise for treating diseases driven by oxidative stress. However, substantial scientific and translational challenges remain, particularly due to the limited understanding of AOE-specific EV sorting mechanisms. These challenges extend across several key dimensions, including large-scale production, enzyme loading efficiency, stability, targeting, characterization, and regulatory issues.

### 6.1. Large-Scale Production and Quality Standards

One major barrier in EV-mediated enzyme delivery is the low yield and high cost associated with current EV isolation methods. Traditional techniques such as ultracentrifugation and density gradient centrifugation generate low yields and require labor-intensive workflows, which cannot meet the demand for clinical applications [108]. Another significant challenge regarding the EV isolation methods lies in the contamination with non-EV-derived proteins, including enzymes. These contaminants may result in false-positive detection of antioxidant enzyme activity, thus complicating the interpretation of experimental outcomes. Furthermore, batch-to-batch heterogeneity poses a significant issue. The EVs isolated from the same cell line show variability in size, membrane proteins, and lipid compositions, which affects the loading efficiency of enzymes and their therapeutic efficacy [109]. Variability also arises from biological factors such as cell state, nutrient composition, and culture stress, all of which reshape EV content. The absence of standardized processes for Good Manufacturing Practice (GMP) production further complicates the clinical translation of EV-based therapies. Currently, no unified purification or sterilization standard is recognized by regulatory agencies [110,111], leaving EV-based therapeutics without a clear manufacturing framework.

Moreover, regarding the detection of EV-derived AOEs, many studies evaluating the enzymatic activity of enzymes delivered by EVs employ insufficiently rigorous methodologies. Due to their low abundance and susceptibility to inactivation, simple enzyme activity assays can be unreliable, thereby obscuring the true biological relevance of the findings. To address these challenges, future research should prioritize the development of standardized protocols for EV isolation to ensure sample purity and consistency. Additionally, more accurate and sensitive assays for evaluating enzymatic activity are essential to obtain reliable and reproducible results.

### 6.2. Enzyme Loading Efficiency and Activity Preservation

Efficient enzyme loading of AOEs into EVs remains difficult. Common techniques such as sonication, extrusion, and electroporation achieve only modest loading efficiency for large enzymes like catalase and SOD, which are insufficient to meet therapeutic dosages [112]. These high-energy techniques can lead to structural damage to the enzymes, decreasing their biological activity. The reproducibility of loading methods is another issue, as identical procedures often yield different results when performed across different labs or batches of EVs [113]. Reported efficiencies vary widely across studies because “loading” is often quantified by total protein rather than by enzyme activity, which frequently overestimates the true functional payload. The stability of the enzyme-EV complex is also a concern, as enzymes might detach from the EVs rapidly in vivo due to non-specific adsorption, potentially leading to the premature clearance of free enzymes by the immune system [113]. Recent studies have shown that MOFs offer a promising solution to enhance the stability and loading efficiency of enzymes into EVs. MOFs, with their tunable porosity and high surface area, can provide a more stable and controlled environment for enzyme encapsulation, improving both loading efficiency and the in vivo retention of the enzyme-EV complex [112,114]. However, their long-term biodegradability, metal-ion residue, and biosafety remain insufficiently evaluated.

### 6.3. Stability and Storage Challenges

EVs are sensitive to freezing, thawing, and mechanical stress, resulting in membrane rupture, aggregation, or cargo loss. EVs, especially when loaded with enzymes, face stability challenges during storage and transportation. Cryopreservation at temperatures as low as −80 °C can cause membrane rupture and aggregation, compromising the protective role of EVs for the enzyme cargo [115]. The storage medium also plays a crucial role, as different buffers and media (e.g., PBS, glycosylation buffers) can significantly affect both the integrity of EVs and the activity of the enzymes encapsulated within them [115]. Additionally, the need for cold-chain logistics to maintain the stability of EVs adds substantial costs, limiting large-scale commercialization and clinical use. The field currently lacks systematic comparisons of storage conditions or mechanistic studies explaining how storage-induced biophysical changes affect in vivo efficacy. Until such evidence emerges, reliable long-term storage of EV-enzyme formulations remains an unmet challenge.

### 6.4. Targeted Delivery and Tissue Penetration

Targeting specific tissues with EVs remains a significant challenge in enzyme delivery. After systemic administration, the majority of EVs are quickly captured by macrophages in the liver and spleen, leading to low accumulation in target tissues like the brain, heart, or eyes [116]. Although surface engineering of EVs with peptides like LAMP2B has been shown to improve brain delivery, efficiency remains suboptimal [117]. Furthermore, the lack of efficient targeting ligands, such as folate receptor or heart-specific peptides, makes tissue-specific targeting costly and inefficient [118]. Additionally, the mechanisms by which EVs enter target cells, whether via fusion, endocytosis, or membrane interaction, are not fully understood, hindering the precise control of delivery efficiency [111]. Another risk involves off-target oxidation or reduction, which can occur if the enzyme is unevenly loaded or if its release is not properly controlled, resulting in oxidative damage in non-target tissues [119]. More mechanistic work is required to define safe and effective targeting strategies.

### 6.5. Analytical Characterization and Quality Control

Characterization of EVs and their enzymatic cargo suffers from a lack of standardized measurement criteria. Various metrics, including size, concentration, protein/lipid composition, and enzyme activity, are measured using different methods, making it difficult to compare results across labs [109,115]. Furthermore, the absence of reference materials for EVs complicates the calibration of instruments and the validation of enzyme loading efficiencies [110]. High-resolution techniques such as flow cytometry, cryo-electron microscopy, and mass spectrometry are often too costly for routine quality control, limiting widespread adoption in EV-based therapeutic development [115]. The absence of reference materials further hinders instrument calibration and assay validation.

### 6.6. Regulatory and Clinical Translation

Regulatory uncertainty remains a major obstacle [111]. Globally, there is no unified classification, approval pathway, or quality standard for EV-based medicines. This regulatory uncertainty complicates clinical trial design and delays market approval. Although some early-phase clinical trials have been conducted, the safety data are limited, and long-term risks, such as immune responses and potential carcinogenesis, have not been systematically evaluated [120]. Additionally, cost-effectiveness is difficult to evaluate, as EV-AOE therapies have not yet demonstrated clear superiority over existing antioxidants or recombinant enzymes. Future clinical development will require well-defined endpoints, standardized dosing strategies, and disease indications where EV delivery provides a measurable advantage.

## 7. Future Directions

Future research on AOE-loaded EVs should aim to deepen mechanistic understanding, optimize therapeutic design, and accelerate clinical translation. To address current mechanistic gaps, future studies should explore how selective AOE packaging occurs at the molecular level, including the roles of ESCRT-dependent pathways, lipid raft-driven sorting, post-translational modifications, and organelle contact sites in cargo recruitment and vesicle formation. Targeted investigations of the intracellular fate of EV-delivered AOEs, including endosomal escape, lysosomal degradation, and cytosolic release, are critically needed. Furthermore, elucidating downstream signaling pathways modulated by EV-borne AOEs, such as effects on redox-sensitive transcription factors (e.g., Nrf2), mitochondrial homeostasis, inflammatory signaling (NF-κB), and cell survival pathways, represents a major unmet need. First, high-resolution proteomic and lipidomic profiling is needed to map the diversity and abundance of antioxidant cargo within EVs derived from different cellular and physiological sources. Such mapping will clarify how oxidative or inflammatory stress influences AOE packaging and release. Integrating these analyses with functional assays and redox-sensitive biosensors could allow real-time tracking of EV function, intracellular antioxidant activity, and downstream signaling in recipient cells, facilitating the establishment of quantitative redox pharmacokinetics. Second, rational engineering approaches, including genetic modification, preconditioning, and biohybrid nanostructure integration, should be employed to enhance both the antioxidant payload and tissue specificity of EVs. Combining MOF-based nanocarriers or synthetic membranes with natural EVs may also improve enzyme stability, loading efficiency, and controlled release. Third, the integration of redox-sensitive biosensors into experimental models could allow real-time tracking of EV function and intracellular antioxidant activity in vivo, facilitating the establishment of quantitative redox pharmacokinetics. Fourth, expanding disease-specific profiling of EVs, particularly in metabolic, inflammatory, and degenerative diseases such as MASLD, cardiovascular disease, and neurodegeneration, could reveal diagnostic and prognostic EV signatures. Finally, advancing clinical translation will require the development of standardized GMP-grade production pipelines, validated analytical methods, and regulatory frameworks. These steps could ultimately enable the use of EVs as a novel form of enzyme replacement therapy for oxidative stress-related disorders.

## 8. Conclusions

EVs represent a natural, biocompatible, and multifunctional platform for the delivery of AOEs. Evidence from stem cell-, immune cell-, and plant-derived EVs highlights their intrinsic ability to modulate redox homeostasis under both physiological and pathological conditions. Engineered EVs further extend these advantages by enabling precise control over cargo composition, targeting, and therapeutic efficacy. Despite current limitations in production scalability, loading efficiency, and clinical standardization, ongoing progress in EV engineering, characterization, and regulatory harmonization holds strong promise. With continued refinement, EV-mediated AOEs delivery could emerge as a next-generation redox-based therapeutic strategy, bridging the gap between natural defense mechanisms and advanced nanomedicine.

## Figures and Tables

**Table 1:** Antioxidant enzymes (AOEs) are reported in EVs.

Antioxidant Enzymes (AOEs) in EVs	EV Source	EV Targeting	Alterations in AOEs-Loading EVs	Detection Methods	Reference
Superoxide Dismutase (SOD)	The media of human dental pulp stem cells (senescent)	Human dental pulp stem cells (young)	EVs **↑** *SOD2* in EVs **↓** under oxidative stress (OR)	qPCR	Mas-Bargues et al., 2023 [20]
	Human dental follicle stem cells (DFSCs)	Periodontal ligament stem cells (PDLSCs)	EVs **↑** SOD1 in EVs **√** under LPS	Proteomics analysis	Huang et al., 2022 [21]
	The cardiac EVs from the mouse heart		SOD1/2/3 in EVs **√**		Claridge et al., 2021 [22]
	Human serum samples and skeletal muscle cells		SOD2 in EVs **↑** after high-intensity interval training (HIIT)		Lisi et al., 2023; Apostolopoulou et al., 2021 [17,23]
	Human blood plasma		SOD1/2 in EVs **√**		McIlvenna et al., 2023 [24]
	Mitochondrial-derived vesicles from rat heart		SOD2 in EVs **√**		Vasam et al., 2021 [25]
	*Chlamydomonas reinhardtii*		SOD in EVs **√**		Garaeva et al. [26]
	Human blood plasma		SOD1 in EVs **─** SOD2 in EVs **↑** after endurance exercise (EEx)	Western blot analysis	Lisi et al., 2023 [17,27]
	Human umbilical cord mesenchymal stem cells (hUC-MSCs)	Hepatocytes; neutrophils	SOD2 in EVs **↓** after knocking down SOD2 in hUC-MSCs		Yao et al., 2019 [28]
	Monocyte-derived macrophages		SOD1 in EVs **↓** after HIV infects cells		Haque et al., 2020 [29]
	Human umbilical cord blood-derived MSCs	Human mononuclear cells (hMNCs); HaCaT Cell; Human Dermal Fibroblast (HDF);	SOD3 in EVs **√** after SOD3 transduction in MSCs		Yang et al., 2020 [30]
	Mouse neutrophils	Mouse liver endothelial cell	EVs **↑** SOD2 in EVs **↑** under LPS		Bao et al., 2022 [31]
	Mouse/human blood plasma	Human umbilical vein endothelial cells (HUVECs)	EVs **↑** SOD3 in EVs **↑** after exercise training (ExT)		Abdelsaid et al., 2022 [32]
	The media of human bone marrow MSCs	Nucleus pulposus cells	SOD1 in EVs **↑**, SOD2 in EVs ─ after MSCs were treated with quercetin		Peng et al., 2024 [19]
	Human neutrophils		SOD in EVs **√**	Western blot analysis; Enzymatic assays	Iversen et al., 2016 [33]
	Supernatant from cell culture media: human peripheral blood mononuclear cells	Monocytic cell lines (U937 cells)	SOD1/2 in EVs **√** SOD 1 **↑** after U937 cells are exposed to cigarette smoke condensate (CSC)	qPCR; Western blot analysis	Haque et al., 2017 [34]
	Plasma of exercise training wild-type mice	The central nervous system and myocardium	EVs **↑** SOD2 in EVs **↑** after exercise training (ExT)	Proteomics analysis; Western blot analysis	Gao et al., 2021 [35]
	Skeletal muscle-derived EVs				
	The adipose tissue from mice	AML12 (alpha mouse liver 12) cells	SOD1 in EVs **ⅹ** SOD activity in EVs **─** under a high-fat diet (HFD)	Enzymatic assays	Jeong et al., 2025 [36]
	*Aloe vera* peels	Human keratinocytes and fibroblasts	SOD activity in EVs **√**		Kim et al. [37]
	Fruits-derived EVs				Logozzi et al. [38]
	Fruits-derived EVs	HDF			Di Raimo et al. [39]
	*Allium cepa*	Mouse skin			Azizi et al. [40]
Catalase (CAT)	The media of human dental pulp stem cells (senescent)	Human dental pulp stem cells (young)	EVs **↑** *CAT* in EVs **─** under OR	qPCR	Mas-Bargues et al., 2023 [20]
	Human blood plasma	Human iPSC-derived cardiomyocytes	CAT activity in EVs **↑** after EEx	Proteomics analysis; Enzymatic assays	Lisi et al., 2023 [41]
	The adipose tissue from mice	AML12	CAT in EVs **↑** CAT activity in EVs **↑** under HFD	Western blot analysis; Enzymatic assays	Jeong et al., 2025 [36]
	Cardiac EVs from the mouse heart		CAT in EVs **√**	Proteomics analysis	Claridge et al., 2021 [22]
	Human serum samples and skeletal muscle cells		CAT in EVs **↑** after HIIT		Lisi et al., 2023; Apostolopoulou et al., 2021 [17,23]
	Human blood plasma		CAT in EVs **√**		McIlvenna et al., 2023 [24]
	*Pseudomonas syringae pv. tomato* DC3000 (*Pto* DC3000)				Deng et al., 2025 [42]
	*Chlamydomonas reinhardtii*				Garaeva et al. [26]
	Human blood plasma		CAT in EVs **↑** after EEx	Western blot analysis	Lisi et al., 2023 [17,27]
	Monocyte-derived macrophages		CAT in EVs **↓** after cells are infected by HIV		Haque et al., 2020 [29]
	Supernatant from cell culture media: human peripheral blood mononuclear cells	Monocytic cell lines (U937 cells)	CAT ↑ after U937 cells are exposed to CSC	qPCR; Western blot analysis	Haque et al., 2017 [34]
	Human serum samples		CAT in EVs **↑** after HIIT	Proteomics analysis; Western blot analysis	Kobayashi et al., 2021 [43]
	Fruits-derived EVs		CAT in EVs **√**	Enzymatic assays	Logozzi et al. [38]
	Fruits-derived EVs	HDF			Di Raimo et al. [39]
Peroxiredoxin (PRDX)	Human serum samples and skeletal muscle cells		PRDX1/2 in EVs **↑** after HIIT	Proteomics analysis	Lisi et al., 2023; Apostolopoulou et al., 2021 [17,23]
	Human blood plasma		PRDX1/2/6 in EVs **√**		McIlvenna et al., 2023 [24]
	Mitochondrial-derived vesicles from rat heart		PRDX3/5/6 in EVs **√**		Vasam et al., 2021 [25]
	Induced pluripotent stem cell (iPSC); mesenchymal stem cell (MSC)		PRDX1/2 in EVs **↓** under progerin-induced senescence	Proteomics analysis; Western blot analysis	Liu et al., 2019 [44]
	Human blood plasma		PRDX1 in EVs **↑** after EEx		Warnier et al., 2025 [45]
	Human serum samples		PRDX2 in EVs **↑** after HIIT		Kobayashi et al., 2021 [43]
	Supernatant from cell culture media: human peripheral blood mononuclear cells	Monocytic cell lines (U937 cells)	PRDX 6 **↓** after U937 cells are exposed to CSC	qPCR; Western blot analysis	Haque et al., 2017 [34]
	The media of human bone marrow MSCs	Nucleus pulposus cells	PRDX1/2 in EVs ─ after MSCs were treated with quercetin	Western blot analysis	Peng et al., 2024 [19]
	Macrophages	Cancer cells	PRDX 6 in EVs **√**		Zheng et al., 2025 [46]
Glutathione Peroxidase (GPX)	The media of human dental pulp stem cells (senescent)	Human dental pulp stem cells (young)	EVs **↑** *GPX* in EVs **↓** under OR	qPCR	Mas-Bargues et al., 2023 [20]
	The adipose tissue from mice	AML12	GPX1 in EVs **ⅹ** GPx in EVs activity ─ under HFD	Enzymatic assays	Jeong et al., 2025 [36]
	Cardiac EVs from the mouse heart		GPX1/3/4/7/8 in EVs **√**	Proteomics analysis	Claridge et al., 2021 [22]
	Human blood plasma		GPX in EVs **√**		McIlvenna et al., 2023 [24]
	Pig seminal plasma exosomes	Sperm	GPX5 in EVs **√**	Proteomics analysis; Western blot analysis	Huang et al., 2024 [47]
	The media of human glioblastoma cell line, GBM8401, and primary glioblastoma cells, GBM04T	Glioblastoma; HUVECs; U251 cells	GPX1 in EVs **↑** under hypoxia	Western blot analysis	Lei et al., 2023 [48]
Glutathione Reductase (GSR)	Human blood plasma	Human iPSC-derived cardiomyocytes	GSR activity in EVs **↑** after EEx	Proteomics analysis; Enzymatic assays;	Lisi et al., 2023 [41]
	DFSCs	PDLSCs	EVs **↑** GSR in EVs **↑** under LPS	Proteomics analysis	Huang et al., 2022 [21]
	Human blood plasma		GSR in EVs **√**		McIlvenna et al., 2023 [24]
Thioredoxin Reductase (TXNRD)	Human blood plasma		TXNRD1 in EVs **─** after EEx	Western blot analysis	Lisi et al., 2023 [17,27]
	Mitochondrial-derived vesicles from rat heart		TXNRD2 in EVs **√**	Proteomics analysis	Vasam et al., 2021 [25]
Thioredoxin Oxidase (TXNRO)	Human blood plasma		TXNRO in EVs **√**	Proteomics analysis	McIlvenna et al., 2023 [24]
	Human blood plasma	The breast cancer cell line MDA-MB-231	TXNRO in EVs **↑** after EEx	Proteomics analysis; Western blot analysis	Sagini et al., 2025 [16]
NAD(P)H dehydrogenase [quinone] 1 (NQO1)	Human blood plasma	The central nervous system and myocardium	EVs **↑** NQO1 in EVs **↑** after ExT	Proteomics analysis; Western blot analysis	Gao et al., 2021 [35]
	Skeletal muscle-derived EVs				
Nicotinamide Phosphoribosyltransferase (NAMPT)	Human blood plasma		NAMPT in EVs **↑** after ExT	Western blot analysis; Enzymatic assays	Lisi et al., 2023; Chong et al., 2022 [17,49]
Heme oxygenase 1 (HMOX1)	Human blood plasma		HMOX1 in EVs **↑** after ExT	Proteomics analysis	Lisi et al., 2023; Bryl-Gorecka., 2018 [17,41,50]
Glucose-6-phosphate dehydrogenase (G6PD)	Human serum samples and skeletal muscle cells		EVs **↑** G6PD in EVs **↑** after HIIT	Proteomics analysis	Lisi et al., 2023; Apostolopoulou et al., 2021 [17,23]
	Human blood plasma		G6PD in EVs **√**		McIlvenna et al., 2023 [24]

Note: increase: “**↑**”; decrease: “**↓**”; no changes: “**─**”; detected: “**√**”; undetected: “**ⅹ**”.

**Table 2:** Representative examples of engineered antioxidant enzyme (AOE)-loaded EVs.

Enzyme Type	EV Source	Assembly Method	Therapeutic Effect	Reference
SOD3	Synovial fibroblast	SOD3 plasmid overexpressing fibroblasts → isolate S-EXOs → load onto polydopamine-coated GelMA microspheres (GM@PDA@S-EXO) for sustained release	Enhance chondrocyte antioxidant capacity, reduce ROS and mitoROS, preserve cartilage extracellular matrix.	Cao et al., 2024 [90]
SOD	Human embryonic kidney cells (HEK293T)	Mechanical extrusion + saponin permeabilization	Reduce ROS level and delay aging in the *C. elegans* model	Shao et al., 2023 [91]
SOD + Chondroitinase ABC	HEK293T	Mechanical extrusion + saponin permeabilization	Reduce ROS, inhibit apoptosis, and promote remyelination in the experimental autoimmune encephalomyelitis mouse model.	Shao et al., 2025 [92]
SOD2	Healthy young human plasma, skimmed milk, and grapes	Sod2-overexpressing plasmid	Grape-derived EV^Sod2^ was the most effective carrier, significantly reducing PM2.5-induced cardiopulmonary injury.	Zhang et al., 2025 [78]
Catalase-SKL	RAW 264.7 macrophages	Sonication	Intranasal administration provides broad brain distribution and no off-target toxicity.	Hayes et al., 2021 [52]
Catalase	J774.A.1 cells	CAT@SiO_2_-ICG (CSI) was transfected into AS1411 aptamer-modified mononuclear macrophage exosomes.	Efficient blood−brain barrier penetration, good cancer-cell-targeting capability, and enhanced sonodynamic therapy of glioblastoma	Wu et al., 2022 [88]
Catalase-Ce6	RAW 264.7 cells (M1 macrophages)	Catalase-Ce6 nanocomplex co-extruded with glucose oxidase-modified M1 macrophage EVs	Cascade oxygenation to enhance photodynamic therapy, efficient tumor targeting, and significant tumor suppression with minimal systemic toxicity	Liu et al., 2021 [93]
Catalase	Mesenchymal stem cells (MSCs)	Encapsulated catalase and MSC-derived exosomes into a blended GelMA/hyaluronic acid hydrogel with thioketal-PEG, forming an O_2_-generating injectable Exo–O_2_ (+) hydrogel	Sustained release of oxygen, which resulted in continuous oxygenation of the metabolically demanding heart cells	Wang et al., 2024 [86]
Catalase & ACSL4 (Acyl-CoA synthetase long-chain family member 4)	4T1 breast cancer cells (engineered via lentiviral overexpression of catalase and ACSL4)	Extraction of exosomes from lentivirus-transfected 4T1 cells and loading of sonosensitizer tetrakis via electroporation to obtain engineered exosomes	Combine oxygen-enhanced sonodynamic therapy and ferroptosis induction	Wu M. et al., 2025 [87]
Catalase	MSCs	Exo/hydrogel loaded with Au nanoparticles and catalase	Oxygen generation to reduce cell apoptosis and necrosis, improve heart cell survival, and promote repair of infarcted cardiac tissue post-myocardial infarction.	Xu et al., 2025, [94]
Catalase	Expi293F cells transfected with the expression constructs	Genetic fusion of proteins (CD9, PhoCl, mCherry, apoptin, catalase)	Light-controlled release of catalase, efficient intracellular delivery for antioxidant and apoptosis-inducing therapy, targeted delivery of proteins to treat liver injury, and induce tumor cell apoptosis	Cheng et al. 2021, [89]
GPX5	HEK293T	Transfect cells with the GPX5 overexpression vector	Enhanced sperm motility, acrosome integrity, reduced oxidative damage, and improved fertilization ability.	Huang et al., 2024 [47]

## Data Availability

No new data were created or analyzed in this study.

## Abbreviations

The following abbreviations are used in this manuscript:

AOE	Antioxidant enzyme
AML12	Alpha mouse liver 12 cells
ALS	Amyotrophic lateral sclerosis
DFSCs	Dental follicle stem cells
ESCRT	Endosomal sorting complex required for transport
EV	Extracellular vesicle
ExT	Exercise training
EEx	Endurance exercise
G6PD	Glucose-6-phosphate dehydrogenase
GMP	Good Manufacturing Practice
GPX	Glutathione peroxidase
GSH	Glutathione
GSR	Glutathione reductase
GST	Glutathione S-transferase
GSSG	Oxidized glutathione
H_2_O_2_	Hydrogen peroxide
HMOX1	Heme oxygenase 1
hUC-MSC	Human umbilical cord–derived mesenchymal stem cell
hMNCs	Human mononu-clear cells
HDF	Human Dermal Fibroblast
HIIT	High-intensity interval training
LPS	Lipopolysaccharide
MASLD	Metabolic dysfunction–associated steatotic liver disease
MEV	Milk-derived extracellular vesicle
Mn-SOD	Manganese superoxide dismutase
MOF	Metal–organic framework
MSC	Mesenchymal stem cell
mTORC1	Mechanistic target of rapamycin complex 1
NADPH	Nicotinamide adenine dinucleotide phosphate (reduced form)
NAMPT	Nicotinamide phosphoribosyltransferase
NQO1	NAD(P)H dehydrogenase [quinone] 1
O_2_^−^•	Superoxide anion
OR	Oxidative stress
PRDX	Peroxiredoxin
PDLSCs	Periodontal ligament stem cells
ROS	Reactive oxygen species
SOD	Superoxide dismutase
SOD1	Cytosolic Cu/Zn-superoxide dismutase
SOD2	Mitochondrial Mn-superoxide dismutase
SOD3	Extracellular superoxide dismutase
TRXNRD1	Thioredoxin reductase 1
TXN	Thioredoxin
TXNRO	Thioredoxin oxidase
